# GDF15 Circulating Levels Are Associated with Metabolic-Associated Liver Injury and Atherosclerotic Cardiovascular Disease

**DOI:** 10.3390/ijms26052039

**Published:** 2025-02-26

**Authors:** Josefa Girona, Montse Guardiola, Emma Barroso, María García-Altares, Daiana Ibarretxe, Núria Plana, Josep Ribalta, Núria Amigó, Xavier Correig, Manuel Vázquez-Carrera, Lluís Masana, Ricardo Rodríguez-Calvo

**Affiliations:** 1 Research Unit on Lipids and Atherosclerosis, University Rovira i Virgili, 43201 Reus, Spain; josefa.girona@urv.cat (J.G.); montse.guardiola@urv.cat (M.G.); daiana.ibarreche@urv.cat (D.I.); nuria.plana@salutsantjoan.cat (N.P.); josep.ribalta@urv.cat (J.R.); luis.masana@urv.cat (L.M.); 2 Vascular Medicine and Metabolism Unit, “Sant Joan de Reus” University Hospital, 43204 Reus, Spain; 3 Pere Virgili Health Research Institute (IISPV), 43007 Tarragona, Spain; namigo@biosferteslab.com; 4 Spanish Biomedical Research Centre in Diabetes and Associated Metabolic Disorders (CIBERDEM), Institute of Health Carlos III, 28029 Madrid, Spain; ebarroso@ub.edu (E.B.); maria.garciaaltares.perez@gmail.com (M.G.-A.); xavier.correig@urv.cat (X.C.); mvazquezcarrera@ub.edu (M.V.-C.); 5 Pharmacology Unit, Department of Pharmacology, Toxicology and Therapeutic Chemistry, Faculty of Pharmacy and Food Sciences, University of Barcelona, 08028 Barcelona, Spain; 6 Institut de Biomedicina de la Universidad de Barcelona (IBUB), University of Barcelona, 08028 Barcelona, Spain; 7 Institut de Recerca Sant Joan de Déu (IR-SJD), 08950 Barcelona, Spain; 8 Metabolomics Platform, Department of Electronic Engineering (DEEEA), University Rovira i Virgili, 43007 Tarragona, Spain; 9 Biosfer Teslab, 43201 Reus, Spain; 10 Department of Basic Medical Sciences, University Rovira i Virgili, 43201 Reus, Spain

**Keywords:** GDF15, MASLD, CV risk, atherosclerotic profile

## Abstract

There is growing evidence linking growth differentiation factor 15 (GDF15) to both metabolic dysfunction-associated steatotic liver disease (MASLD) and cardiovascular (CV) risk. Nevertheless, the potential relationship between circulating levels of GDF15 and key features of MASLD being predisposed to atherosclerotic CV disease is not fully unveiled. The aim of this study was to deepen into the role of circulating GDF15 levels on metabolic-associated liver injury and atherosclerotic CV disease. We determined the serum GDF15 levels in 156 participants of a metabolic patient-based cohort, and cross-sectionally explored its associations with liver injury and an advanced atherosclerotic lipoprotein profile assessed by nuclear magnetic resonance (^1^H-NMR). Additionally, we prospectively evaluated the association between GDF15 levels at baseline and incident atherosclerotic CV disease after a 10-year follow-up. GDF15 was related to liver injury and inflammatory hallmarks, and it increased the likelihood for liver steatosis independently of confounding factors. Likewise, GDF15 was positively associated with an atherogenic profile, particularly with the number of very-low-density lipoproteins (VLDL) particles and its cholesterol and triglyceride content, and with an indicator of subclinical atherosclerosis (i.e., carotid intima–media thickness (cIMT)). The baseline serum GDF15 levels were higher in the patients with atherosclerotic CV disease (10.6%) after a 10-year follow-up than in the individuals without CV disease. Altogether, this study provides new insights into the role of GDF15 in both MASLD and CV disease.

## 1. Introduction

Liver ectopic fat accumulation is the main feature of the so-called metabolic dysfunction-associated steatotic liver disease (MASLD), the leading cause of chronic liver disease worldwide [1,2,3,4]. In fact, it is estimated that one in four people around the world are affected by this pathology in the absence of alcohol abuse [5], reaching a prevalence higher than 50% in individuals with metabolic-related disorders [3,6,7,8,9].

Liver fat accumulation is due to both an increase in the non-esterified fatty acids uptake and in triglyceride de novo synthesis, thereby exceeding the capacity of fat removal via very-low-density lipoprotein (VLDL) secretion [6,10]. Therefore, the course of MASLD may parallel a pro-atherogenic plasma lipoprotein profile, mainly characterized by an increase in VLDL production, aimed at counteracting the excessive fat depots in the liver. Together with low-density lipoproteins (LDL), increasing evidence highlights the role of VLDL as a major contributor to atherosclerotic risk [11,12]. Since the leading cause of death among MASLD patients is cardiovascular (CV) disease [13,14], the MASLD-associated atherogenic lipoprotein profile may underlie the increased CV risk in these patients.

MASLD ranges in severity from the metabolic dysfunction-associated fatty liver (MAFL) to the metabolic dysfunction-associated steatohepatitis (MASH), characterized by an increased risk of liver fibrosis in its advanced stages [15]. MASH can irreversibly progress to end-stage liver disease, including cirrhosis and hepatocellular carcinoma [7]. Nevertheless, despite the comprehensive guidelines for the assessment and management of MASLD [16], early detection is poor, since most patients do not report symptoms until they have progressed to advanced stages. Imaging techniques for the screening of large populations are unreliable and specific serum biomarkers are lacking, with the highly invasive liver biopsy being the gold standard for MASLD diagnosis (for review see [17,18,19,20]). Therefore, the identification of new molecules useful for the diagnosis and monitoring of the disease is of utmost importance.

Growth differentiation factor 15 (GDF15) is a stress-response cytokine that has recently been related to several metabolic conditions [21,22], including chronic liver diseases [23,24,25]. The mature-form GDF15 is released from different tissues to the bloodstream as a 25 kDa homodimer in response to different stresses, such as metabolic disorders and cardiovascular diseases, among other pathological conditions. Circulating GDF15 binds to glial cell line-derived neurotrophic factor (GDNF) family receptor α-like (GFRAL), thereby acting as a metabolic regulator, improving glucose tolerance and insulin sensitivity, and controlling food intake and body weight (for review see [26]). Thus, GDF15 actively contributes to the maintenance of metabolic homeostasis [27]. Circulating GDF15 levels have been found to increase in patients with MASH compared to healthy controls or patients without simple steatosis [25]. Specifically, systemic GDF15 concentrations increase with hepatic fibrosis and correlate with liver stiffness, measured by elastography [25]. Additionally, circulating levels of GDF15 have been related to atherosclerosis and CV disease (for review see [28]). Nevertheless, the potential relationship between circulating levels of GDF15 and key features of MASLD being predisposed to atherosclerotic CV disease, such as an altered atherogenic profile, has not been fully explored.

Here, we analyzed the circulating levels of GDF15 in patients with metabolic disorders to explore their potential relationship with baseline MASLD and the incidence of CV disease after a 10-year follow-up.

## 2. Results

### 2.1. Characteristics of Study Population

The characteristics of the study population are shown in Table 1.

Age, blood pressure, weight, waist circumference, BMI, total cholesterol and glucose were higher in the patient group. Additionally, hallmarks of liver injury, including AST, ALT, GGT, HsCRP, the acute-phase proteins Glyc-A and Glyc-B, as well as the non-invasive scores for liver fibrosis (FIB-4) and steatosis (FLI), were higher in the patients compared to the healthy volunteers. Diabetes, obesity, metabolic syndrome, and hypertension were more present in the patients group. Accordingly, the percentage of individuals receiving insulin, oral antidiabetic drugs and hypotensive therapy was higher in the patients with metabolic disturbances. Additionally, the percentage of individuals with liver steatosis (i.e., FLI ≥ 60) was also higher in the patients than in the volunteers group.

**Figure 1:**
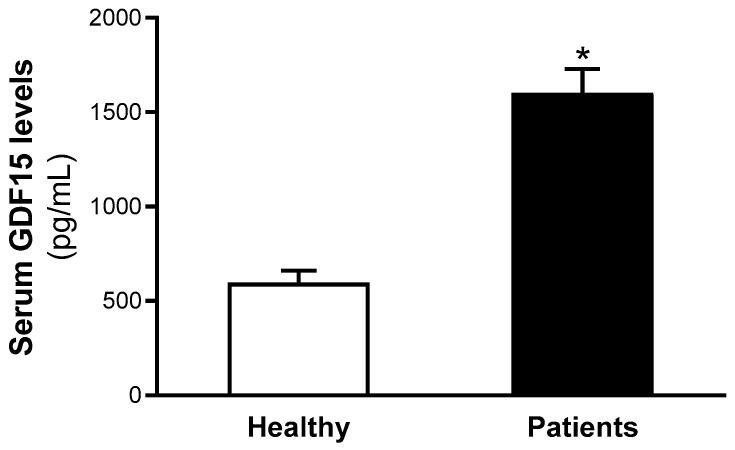
Serum GDF15 levels increased in patients with metabolic disturbances. Data are expressed as the means ± SEM. * *p* < 0.05 vs. healthy volunteers. *p* values are adjusted by age, insulin therapy, oral antidiabetic therapy and hypotensive therapy through the analysis of covariance (ANCOVA).

Furthermore, increased GDF15 levels were found in the serum of the patients compared with healthy individuals (healthy: 503.5 [371.2–703.8] pg/mL; patients: 1204.9 [798.2–1876.5] pg/mL, *p* < 0.05) (Figure 1).

### 2.2. Serum GDF15 Is Associated with Liver Injury and Inflammation Hallmarks

The potential relationship between serum GDF15 and hallmarks of liver injury (i.e., AST, ALT and GGT), inflammation (HsCRP and acute-phase glycoproteins, such as Glyc-A and Glyc-B) and non-invasive scores for liver steatosis (FLI) and fibrosis (FIB4) were analyzed (Table 2).

GDF15 was found positively correlated with ALT, GGT, HsCRP, Glyc-A, Glyc-B, FLI and FIB4. After adjusting for confounding factors correlations between GDF15 and GGT, Glyc-A, FIB4 and FLI remained statistically significant. No significant correlations were found between GDF15 and AST.

**Figure 2:**
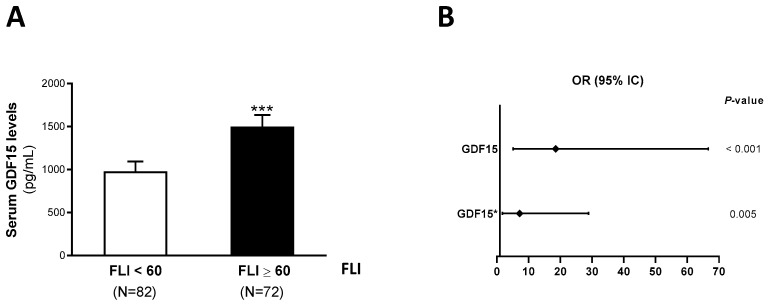
(**A**) Serum GDF15 levels increased in subjects with liver steatosis. Serum GDF15 levels are shown in patients with FLI ≥ 60 or those with FLI < 60. Data are expressed as the means ± SEM. *** *p* < 0.001 vs. FLI < 60 individuals. (**B**) Univariate and multivariate logistic regression models (odds ratio, OR and 95% confidence interval, CI) were used to explore the associations between serum GDF15 and liver steatosis as outcomes. * Multivariate logistic regressions models were adjusted for age, insulin therapy, oral antidiabetic therapy and hypotensive therapy (METOD = Wald).

Next, the population was stratified in those with FLI ≥ 60 and those with FLI < 60, with liver steatosis being defined as FLI ≥ 60 [29]. The serum GDF15 levels were significantly increased in patients with FLI ≥ 60 compared with those with FLI < 60 (FLI < 60: 800.0 [571.3–1476.5] pg/mL; FLI ≥ 60: 1167.6 [799.1–1615.4] pg/mL, *p* < 0.001) (Figure 2A). Then, univariate and multivariate logistic binary regression models were performed to assess the risk of liver steatosis based on the serum GDF15 levels. Serum GDF15 was positively related to liver steatosis in the crude model (odds ratio [OR], 95% confidence interval [CI] = 18.55 (5.16–66.65), *p* < 0.001). In the model including age, insulin therapy, oral antidiabetic therapy and hypotensive therapy, the associations between GDF15 and liver steatosis remained statistically significant (odds ratio [OR], 95% confidence interval [CI] = 7.21 (1.80–28.91), *p* = 0.005) (Figure 2B).

### 2.3. Associations Between Serum GDF15 and CV Risk

The advance atherogenic profile was determined using the Liposcale test^®^ (Biosfer Teslab, Reus, Spain) (Supplemental Table S1). The relationships between serum GDF15 and the advanced lipid profile, including the lipid concentrations of triglycerides (TG) and cholesterol (C), the size (Z) and concentration of particles (P) of three different lipoproteins (VLDL, LDL and HDL) and the concentration of particles (P) of nine subclasses (large, medium and small VLDL, LDL and HDL) were analyzed (Table 3). Serum GDF15 was positively associated with the cholesterol content of VLDL and LDL, and inversely associated with the cholesterol content of HDL. Additionally, serum GDF15 positively correlated with the triglyceride content of the three lipoproteins (VLDL, LDL, HDL) and with the number of particles of VLDL (total, large, medium, small), LDL (total, large, medium, small), and large HDL. An inverse correlation was found between serum GDF15 and LDL size. After adjusting for age, insulin therapy, oral antidiabetic therapy, and hypotensive therapy, GDF15 remained directly associated with the cholesterol content of VLDL and the triglyceride content of both VLDL and LDL. Additionally, the association between serum GDF15 and the number of particles of VLDL (total, large, medium, small) remained statistically significant after adjusting for the abovementioned confounding factors.

**Figure 3:**
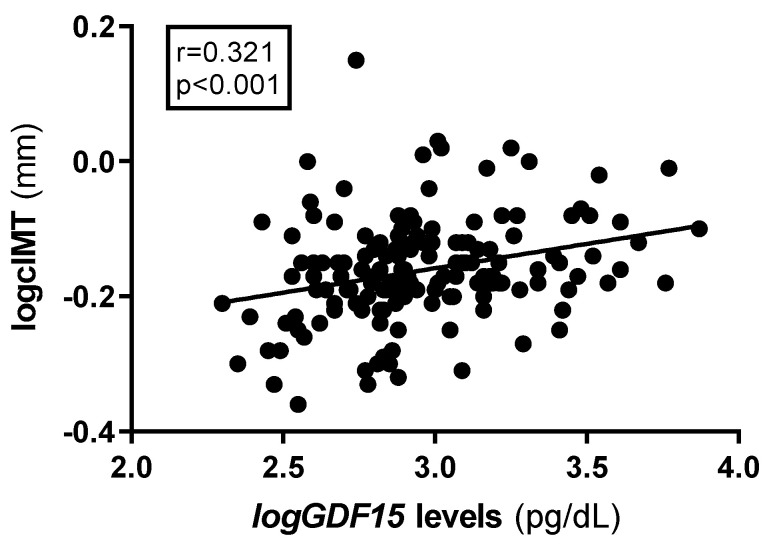
Plots of plasma correlations between GDF15 and cIMT. r from rho coefficients from the Spearman correlation analysis. GDF15 and cIMT was log-transformed to reduce skewness. GDF15: growth differentiation factor 15; cIMT: carotid intima–media thickness.

Spearman bivariate correlation analysis showed positive associations between GDF15 and carotid intima–media thickness (cIMT) (Figure 3).

**Figure 4:**
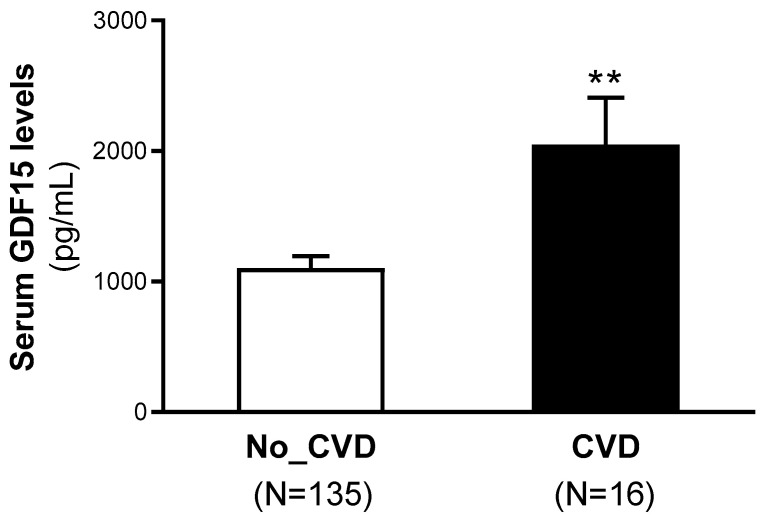
Baseline serum GDF15 levels of the patients according to cardiovascular (CV) disease after a 10-years follow-up. Data are expressed as the means ± SEM. ** *p* < 0.01 vs. non-CV disease patients.

Finally, data on the development of atherosclerotic CV disease after a 10-year follow-up was recorded. During the follow-up period, two individuals were lost due to transfer to an autonomous community other than Catalonia. From the rest of the participants, one individual died due to CV disease and six due to other causes, including cancer, infectious disease and post-surgical complications. Nevertheless, since four of them developed CV disease before dying, they were included in the study. A total of 16 individuals without previous clinical history developed atherosclerotic CV disease during this period (10.6% incidence). The baseline serum GDF15 levels were higher in the patients with atherosclerotic CV disease after a 10-year follow-up than in the individuals without CV disease (non-CVD: 752.6 [508.8–1261.5] pg/mL; CVD: 1599.1 [970.9–2921.2] pg/mL, *p* < 0.01) (Figure 4).

## 3. Discussion

MASLD presents as asymptomatic until advanced irreversible stages, and thus, emerging non-invasive methods for its early diagnosis and monitoring beyond the liver biopsy are eagerly awaited. Although GDF15 has been related to chronic liver diseases [23,24,25], including some aspects of MASH such as hepatic fibrosis and liver stiffness [25], the potential relationship between circulating levels of GDF15 and key features of MASLD predisposing to atherosclerotic CV disease has not been fully explored.

As mentioned above, circulating levels of GDF15 have been previously related to chronic liver diseases [23,24,25]. Our data show that both serum GDF15 levels and liver injury hallmarks were higher in patients with metabolic disturbances compared with healthy volunteers. Accordingly, GDF15 was positively correlated with GGT, but not with AST or with ALT, after adjusting by age, insulin therapy, oral antidiabetic therapy and hypotensive therapy. However, it is worth noting that both transaminases may not be reliable markers of the disease, since just 25% of MASLD patients show elevated transaminases levels [30]. Despite GDF15 was not associated with HsCRP after adjustment for confounding factors, it positively correlated to the liver-derived inflammatory hallmarks Glyc-A and Glyc-B. Unlike HsCRP, glycoproteins such as Glyc-A and Glyc-B integrate both the protein levels and the bond aggregation state of several of the most abundant acute-phase proteins in serum, thereby acting as more reliable low-grade inflammation biomarkers than just one single molecule [31]. On the other hand, GDF15 has been related to fibrosis and liver stiffness in MASH patients with proven biopsy [25]. This is supported by other studies showing an association between GDF15 and an increased risk for advanced liver fibrosis [32]. Accordingly, our data showed a positive correlation between GDF15 and FIB-4, even after adjusting with cofounding factors. Concerning the relationship between GDF15 and liver steatosis, somewhat conflicting data have been reported. In a study using two different datasets, GDF15 expression was only negatively correlated with steatosis grade and percentage in one of them, whereas no significant associations were found in the other one [33]. Nevertheless, serum GDF15 levels had been found to increase with the histological severity of MASLD patients, although they were not associated with the steatosis grade [25]. Conversely, another study did not show differences in the serum GDF15 levels between non-MASLD individuals and patients with simple MASLD, although increased GDF15 was associated with an increased risk of simple MASLD [32]. Our data are in line with those studies reporting an increase in serum of GDF15 levels in individuals with liver steatosis, showing that this stress-response cytokine was independently associated with the likelihood of liver steatosis in logistic regression analysis.

CV disease is the leading cause of mortality among MASLD patients [13,14], even higher than liver-related mortality [34]. Since MASLD patients show increased VLDL production, an MASLD-associated atherosclerotic profile may underlie the increased CV risk in these patients. Given the role of GDF15 in both MASLD [35] and CV disease [28], we further explored the potential relationships between this stress-response cytokine and an advanced lipoprotein profile analyzed by ^1^H-NMR. After adjusting for confounding factors, serum GDF15 positively correlated to both cholesterol and triglyceride content of VLDL, and to triglyceride content of LDL. Furthermore, it was associated with the total number of VLDL particles, as well as with the large, medium and small VLDL particles. These findings are in line with a recent study showing positive correlations of GDF15 and VLDL particle number [36]. Although the mechanisms by which VLDL are related to the atherosclerosis physiopathology are still unclear, these lipoproteins have been proposed as a causal risk factor for atherosclerotic CV disease, showing a similar atherogenic risk per particle than LDL [11,12,37]. Therefore, GDF15 could be involved in some way in the liver production of these lipoproteins, thereby acting as a potential agent for the increased CV risk found in the MASLD patients. Actually, GDF15 positively correlated to cIMT, and its basal levels were found statistically higher in patients developing incident atherosclerotic CV disease after a 10-years follow-up period. The role of GDF15 in CV disease is further supported by a recent study reporting an association between subclinical atherosclerosis and a GDF15 single-nucleotid polymorphism (SNP) related to increased circulating levels of this stress-response cytokine [38]. Hence, our data strongly support the role of GDF15 in both MASLD and CV disease.

Some limitations in our study should be acknowledged. Liver biopsies for the diagnosis of liver disease were not available and liver steatosis was assessed by a non-invasive score [29]. Nevertheless, FLI is an algorithm which is highly correlated with the amount of liver fat measured by magnetic resonance spectroscopy [39], that is widely used in epidemiological studies [29,40]. On the other hand, the study was performed in a subset of a population attending a single center. Although our study cohort contains a large percentage of patients with metabolic disturbances, the relatively small sample size may attenuate the impact of the results, and further studies in larger independent populations need to be carried out to validate our findings. Additionally, the cross-sectional nature of our study precludes establishing causal relationships between the serum GDF15 and the outcomes studied. However, data from others strongly support our conclusions [36]. Although the 10-year follow-up period strengthens our findings, the prospective study includes a limited sample size in the group of patients developing atherosclerotic CV disease. Nevertheless, it is worth noting that our findings are in line with other studies showing the relationship with both MASLD [25,32] and CV risk [28]. Here, we add a new puzzle piece, highlighting the relationship between GDF15 and an advanced atherosclerotic profile associated with both MASLD and an increased CV risk.

## 4. Materials and Methods

### 4.1. Study Population

A baseline cross-sectional study and a 10-year follow-up prospective study were performed. A subset of 98 patients from a well-characterized cohort recruited from November 2009 to February 2012 in the Vascular Medicine and Metabolism Unit from the Sant Joan de Reus University Hospital (Reus, Spain) due to lipid metabolism disturbances and other metabolic disorders (i.e., obesity, metabolic syndrome, diabetes or cardiovascular disease) was used [38,41]. A wash-out period of at least 6 weeks was performed by individuals taking lipid-lowering therapies (8 weeks if taking fibrates). Individuals with known serious diseases, including cancer or chronic liver, renal or lung disease were excluded of the study. Additionally, patients with viral or autoimmune hepatic diseases were excluded when these were clinically indicated. Alcohol abuse (the consumption of above 20 g/day in females or above 40 g/day in males) was also considered as an exclusion criterion. A total of 58 healthy volunteers free of metabolic disorders were used as controls.

A 10-year follow-up study was conducted for incident atherosclerotic CV disease. An annual control visit was carried out on all patients. Additionally, after 10 years, a critical review of the clinical history of both patients and volunteers was carried out to identify new atherosclerotic CV events. Subjects who died without developing CV disease and dropouts before ten years were excluded from follow-up analyses. Atherosclerotic CV disease was defined as myocardial infarction, cerebrovascular disease or peripheral artery disease. Specifically, the diagnosis criteria for myocardial infarction and cerebrovascular disease were the presence of acute coronary syndrome or ischemic stroke, respectively, needing hospitalization and with an unequivocal diagnosis in the discharge report. For the diagnosis of peripheral artery disease, both vascular affection by imaging studies and clinical intermittent claudication with the ankle–brachial index (ABI) < 0.9 were used. The ABI was calculated by dividing the blood pressure in the artery in the ankle by the blood pressure in the artery in the arm.

All participants provided written informed consent. The study was approved by the reference Ethic Committees and performed according to the guidelines of the Declaration of Helsinki (approval code: 093/2021, date: 2 June 2021).

### 4.2. Clinical Data and Biochemical Determinations

Demographic, anthropometric and clinical data were recorded at the point of study inclusion. Presence of diabetes, obesity, metabolic syndrome and hypertension was defined in compliance with well-established criteria, as previously described [41].

Venous blood sample were obtained after overnight fasting and was centrifuged at 1500× *g* for 15 min at 4 °C to obtain serum. Serum samples were aliquoted and stored at −80 °C until analysis. Biochemical parameters, including total cholesterol, glucose, the high-sensitive C-reactive protein (HsCRP), transaminases [aspartate aminotransferase (AST) and alanine aminotransferase (ALT)] and gamma-glutamyl transpeptidase (GGT) were measured using turbidimetric or enzymatic methods (Spinreact, SA, Barcelona, Spain) adapted to the Cobas Mira Plus Autoanalyser (Roche Diagnostics, Barcelona, Spain) [41].

Carotid ultrasound imaging by using a MyLab 50-X Vision sonographer (Esaote, Genova, Italy) were performed to determine the carotid intima media thickness (cIMT) in the far wall of both right (RCCA) and left common carotid arteries (LCCA), by using a 7.5 MHz linear array and semiautomated software. The cIMT value was determined by averaging values of both territories. Additionally, bifurcations and internal carotids were also analyzed through a manual method. According to the Mannheim consensus [42], plaques were defined as focal structures with thickness >1.5 mm or protrusions into the lumen by at least 0.5 mm or 50% thicker than the surrounding cIMT.

### 4.3. Non-Invasive Scores for Liver Steatosis and Fibrosis

Fatty liver and liver fibrosis were estimated by the fatty liver index (FLI) and Fibrosis-4 (FIB-4) algorithms, respectively, as indicated elsewhere [43]. Liver steatosis was defined as FLI ≥ 60 [29].

### 4.4. Serum GDF15 Determination

The serum GDF15 levels were assessed using a commercial sandwich enzyme-linked immunosorbent assay kit (Biovendor, Brno, Czech Republic), following the manufacturer’s instructions. The serum GDF15 levels were measured using a standard curve constructed with the kit’s standards.

### 4.5. Glycoprotein Analysis

Acute-phase glycoproteins Glyc-A and Glyc-B were assessed by nuclear magnetic resonance (^1^H-NMR), as previously described in [43].

### 4.6. Advanced Lipoprotein Profile

The advanced lipoprotein profiling, including lipid concentrations of triglycerides (TG) and cholesterol (C), the size (Z) and concentration of particles (P) of 3 different lipoproteins (VLDL, LDL and HDL) and the number of particles (P) of 9 subclasses (large, medium and small VLDL, LDL and HDL) was analyzed using the Liposcale test^®^ [44]. Briefly, particle concentration and diffusion coefficients were obtained from the measured amplitudes and attenuation of their spectroscopically distinct lipid methyl group NMR signals using the 2D diffusion-ordered ^1^H NMR spectroscopy (DSTE) pulse. The methyl signal was surface fitted with 9 lorentzian functions associated with each lipoprotein subclasses: large, medium and small of the main lipoprotein classes. The area of each lorentzian function was related to the lipid concentration of each lipoprotein subclass, and the size was calculated from their diffusion coefficient. Each subclass particle concentration was calculated by dividing the lipid volume by the particle volume of a given class. Lipid volumes were determined by using common conversion factors to convert concentration units into volume units [45]. Finally, weighted average VLDL, LDL and HDL particle sizes were calculated from various subclass concentrations by summing the known diameter of each subclass multiplied by its relative percentage of subclass particle number.

### 4.7. Statistical Analysis

The normality of continuous variables was determined by the Kolmogorov–Smirnov test, and variables not showing normal distributions were log-transformed to reduce skewness. Categorical variables are expressed as frequencies and continuous variables as the median and interquartile range, unless otherwise indicated. Differences between metabolic patients and non-metabolic volunteers were analyzed by Student’s *t*-test, U-Mann–Whitney test or Chi-square tests, as appropriate. The differences between the groups were adjusted by confounding factors through the analysis of covariance (ANCOVA). Correlations between two variables were performed by using Spearman’s rho coefficient (ρ). The study population was stratified in those with FLI ≥ 60 and those with FLI < 60 [29]. Univariate and multivariate logistic binary regression models were performed for dichotomous variables to assess the risk of liver steatosis based on the serum GDF15 levels. The results are presented as odds ratios (OR) and 95% confidence intervals (CI). Statistical analyses were performed using SPSS software (IBM SPSS Statistics, version 22.0). Differences were considered statistically significant at *p* < 0.05.

## 5. Conclusions

Altogether, our study provides new insights on the relationship between MASLD and CV risk, focusing in the role of GDF15 in both pathologies. Given the relationships of GDF15 with both the fatty liver and the advanced atherosclerotic profile, specifically with the number of particles VLDL and its cholesterol and triglyceride content, this stress-response cytokine may be involved in the underlying mechanisms linking both pathologies, although further molecular studies are warranted in order to fully clarify the causative role of GDF15 in the CV risk related to MASLD.

## Figures and Tables

**Table 1:** Baseline characteristics of the study cohort.

	Healthy Volunteers (N = 58)	Patients (N = 98)	*p*-Value
Clinical Data			
Age (years)	48.5 (39.8–53.3)	62.0 (53.4–68.0)	<0.001
Gender (F)	58.6%	50%	0.229
Hypertension	0%	56.1%	<0.001
Diabetes	0%	72.4%	<0.001
Obesity	0%	56.1%	<0.001
Metabolic syndrome	0%	91.8%	<0.001
Steatosis	1.8%	73.2%	<0.001
Anthropometric and analytical data			
Systolic BP (mmHg)	115.0 (103.8–125.0)	136.0 (129.5–150.0)	<0.001
Diastolic BP (mmHg)	76.0 (70.0–80.0)	79.0 (72.5–85.0)	0.021
Weight (Kg)	65.9 (61.4–78.4)	83.0 (74.3–94.2)	<0.001
Waist circumference (cm)	84.0 (79.0–91.0)	103.0 (97.0–112.0)	<0.001
BMI (Kg/m^2^)	24.9 (23.1–26.8)	30.4 (28.6–33.6)	<0.001
Total cholesterol (mmol/L)	5.1 (4.5–5.6)	6.2 (5.0–7.3)	<0.001
Glucose (mg/dL)	85.1 (79.8–91.2)	132.0 (105.8–157.0)	<0.001
AST (U/L)	21.0 (19.0–24.3)	23.0 (20.8–29.3)	0.004
ALT (U/L)	13.0 (10.0–19.0)	20.0 (14.0–26.5)	<0.001
GGT (U/L)	16.0 (13.0–20.3)	24.0 (16.8–40.3)	<0.001
HsCRP (mg/L)	1.1 (0.5–1.7)	2.3 (1.4–3.1)	<0.001
Glyc-A (µmol/L)	587.1 (523.1–679.0)	957.4 (807.1–1137.8)	<0.001
Glyc-B (µmol/L)	292.3 (258.4–336.3)	371.2 (333.6–417.4)	<0.001
FIB-4	1.4 (1.2–1.6)	1.6 (1.4–2.0)	<0.001
FLI (%)	12.6 (6.1–28.8)	81.8 (58.7–93.5)	<0.001
Treatments			
Insulin therapy	0%	16.3%	0.001
Oral antidiabetic therapy	0%	51.0%	<0.001
Hypotensive therapy	0%	55.1%	<0.001

Data are shown as frequencies or median (interquartile range). Normal distributions were analyzed by Student’s *t*-test, non-normal distribution by U-Mann–Whitney test, and data were gathered as categorical variables by Chi-square tests. Systolic BP: systolic blood pressure; diastolic BP: diastolic blood pressure; BMI: body mass index; AST: aspartate aminotransferase; ALT: alanine aminotransferase; GGT: gamma-glutamyl transferase; HsCRP: high-sensitivity C-reactive protein; FIB-4: fibrosis-4 score; FLI: fatty liver index.

**Table 2:** Relationships between GDF15 and liver injury hallmarks.

Variables	ρ (N = 156)	*p*-Value
AST	0.069	0.391
ALT	0.195	0.015
GGT	0.259	0.001 *
HsCRP	0.345	<0.001
Glyc-A	0.544	<0.001 *
Glyc-B	0.450	<0.001 *
FIB-4	0.434	<0.001 ^#^
FLI	0.543	<0.001 ^#^

Spearman correlations. Significance (*p*-values) of rho coefficients (ρ) between serum GDF15 and liver injury hallmarks in the whole cohort (N = 156). GDF15, AST, ALT, GGT and FLI were log-transformed to reduce skewness. Remained significance after adjustment by age, insulin therapy, oral antidiabetic therapy and hypotensive therapy (*) or by insulin therapy, oral antidiabetic therapy and hypotensive therapy (#). GDF15: growth differentiation factor 15; AST: aspartate aminotransferase; ALT: alanine aminotransferase; GGT: gamma-glutamyl transferase; HsCRP: high-sensitivity C-reactive protein; FIB-4: fibrosis-4 score; FLI: fatty liver index.

**Table 3:** Relationships between serum GDF15 and advanced atherogenic lipid profile.

Variables	ρ (N = 156)	*p*-Value
VLDL-C	0.475	<0.001 *
LDL-C	0.217	0.007
HDL-C	−0.273	0.001
VLDL-TG	0.478	<0.001 *
LDL-TG	0.503	<0.001 *
HDL-TG	0.327	<0.001
VLDL-P	0.474	<0.001 *
Large VLDL-P	0.430	<0.001 *
Medium VLDL-P	0.500	<0.001 *
Small VLDL-P	0.473	<0.001 *
LDL-P	0.296	<0.001
Large LDL-P	0.248	0.002
Medium LDL-P	0.202	0.012
Small LDL-P	0.343	<0.001
HDL-P	−0.070	0.386
Large HDL-P	0.283	<0.001
Medium HDL-P	−0.044	0.586
Small HDL-P	−0.074	0.360
VLDL-Z	0.014	0.860
LDL-Z	−0.170	0.035
HDL-Z	0.074	0.358

Spearman correlations. Significance (*p*-values) of rho coefficients (ρ) between serum GDF15 and the advanced atherogenic profile in the whole cohort (N = 156). GDF15, VLDL-C, VLDL-TG, HDL-TG, VLDL-P, large VLDL-P, medium VLDL-P, small VLDL-P, HDL-P, large HDL-P and medium HDL-P were log-transformed to reduce skewness. * Remained significance after adjustment by age, insulin therapy, oral antidiabetic therapy and hypotensive therapy. GDF15: Growth differentiation factor 15; VLDL-C: very-low-density lipoproteins–cholesterol; LDL-C: low-density lipoproteins–cholesterol; HDL-C: high-density lipoproteins–cholesterol; VLDL-TG: very-low-density lipoproteins–triglycerides; LDL-TG: low-density lipoproteins–triglycerides; HDL-TG: high-density lipoproteins–triglycerides; VLDL-P: very-low-density lipoproteins particles; LDL-P: low-density lipoproteins particles; HDL-P: high-density lipoproteins particles; VLDL-Z: very-low-density lipoproteins size; LDL-Z: low-density lipoproteins size; HDL-Z: high-density lipoproteins size.

## Data Availability

The data presented in this study will be provided by the corresponding author after reasonable inquiry.

## Supplementary Materials

The following supporting information can be downloaded at: https://www.mdpi.com/article/10.3390/ijms26052039/s1.
