# Supplementary material for: GDF15 Circulating Levels Are Associated with Metabolic-Associated Liver Injury and Atherosclerotic Cardiovascular Disease
Source: Int J Mol Sci. 2025 Feb 26;26(5):2039. doi: 10.3390/ijms26052039 (PMC11900571; doi:10.3390/ijms26052039)
Supplement: Supplementary file 1 [file ijms-26-02039-s001.zip › ijms-3423131-supplementary.pdf]

Table S1. Advanced atherogenic lipid profile.

Variables	(N=156)
VLDL-C (mg/dL)	18.4 (9.9-34.0)
LDL-C (mg/dL)	139.4 (116.0-159.4)
HDL-C (mg/dL)	55.9 (50.0-62.8)
VLDL-TG (mg/dL)	70.8 (41.0-125.6)
LDL-TG (mg/dL)	15.9 (12.7-20.8)
HDL-TG (mg/dL)	14.1 (11.0-17.7)
VLDL-P (nmol/L)	52.9 (29.7-90.4)
Large VLDL-P (nmol/L)	1.3 (0.8-2.1)
Medium VLDL-P (nmol/L)	6.3 (3.7-12.1)
Small VLDL-P (nmol/L)	46.1 (25.5-76.7)
LDL-P (nmol/L)	1380.5 (1183.1-1611.3)
Large LDL-P (nmol/L)	220.6 (192.0-243.1)
Medium LDL-P (nmol/L)	410.9 (306.3-498.8)
Small LDL-P (nmol/L)	757.5 (633.8-900.9)
HDL-P (nmol/L)	28.5 (25.5-31.2)
Large HDL-P (nmol/L)	0.3 (0.3-0.4)
Medium HDL-P (nmol/L)	10.3 (9.3-11.9)
Small HDL-P (nmol/L)	17.5 (15.4-20.0)
VLDL-Z (nm)	42.3 (42.2-42.4)
LDL-Z (nm)	21.1 (20.9-21.2)
HDL-Z (nm)	8.3 (8.2-8.4)

Data are shown as median (interquartile range). VLDL-C: very low-density lipoproteins-cholesterol; LDL-C: low-density lipoproteins-cholesterol; HDL-C: high-density lipoproteins-cholesterol; VLDL-TG: very low-density lipoproteins-triglycerides; LDL-TG: low-density lipoproteins-triglycerides; HDL-TG: high-density lipoproteins-triglycerides; VLDL-P: very low-density lipoproteins-particles; LDL-P: low-density lipoproteins-particles; HDL-P: high-density lipoproteins-particles; VLDL-Z: very low-density lipoproteins-size; LDL-Z: low-density lipoproteins-size; HDL-Z: high-density lipoproteins-size.
